# Regulation of allergies across the body by microbial metabolites

**DOI:** 10.1038/s12276-026-01642-1

**Published:** 2026-02-18

**Authors:** Chang H. Kim, James R. Baker

**Affiliations:** 1 Mary H. Weiser Food Allergy Center, Ann Arbor, MI USA; 2 Department of Pathology, University of Michigan School of Medicine, Ann Arbor, MI USA; 3 Department of Medicine, University of Michigan School of Medicine, Ann Arbor, MI USA

**Keywords:** Allergy, Allergy

## Abstract

Allergies are adverse immune responses to typically harmless substances, known as allergens. While allergies can involve diverse immune responses, type 2 immune responses that induce acute hypersensitivity mediated by mast cells, eosinophils and basophils are the major mechanisms underlying allergic disorders. Allergic diseases include atopic dermatitis, allergic rhinitis, food allergies and asthma. The onset and persistence of allergic disorders are influenced by genetic factors, pre-existing illnesses, age, environmental conditions and other lifestyle factors. In particular, diet and microbiomes considerably affect the incidence of various allergic diseases in the skin, lung and intestine. Individuals prone to develop allergic diseases often have impaired and skewed microbial diversification over the first year of life, and this can lead to altered levels of microbial metabolites in the intestine and inflamed tissues. Microbial metabolites, such as short-chain fatty acids, indole metabolites and bile acids, can exert specific regulatory effects on the various components of the immune system, such as barrier epithelial cells and immune cells, including dendritic cells, macrophages, T cells, B cells, innate lymphoid cells and mast cells. Microbial metabolites can also promote immune tolerance to allergenic substances by strengthening regulatory T cells. Understanding the role of these metabolites can lead to better prevention and control of allergic diseases. Here, in this review, we examine current research progress on the interactive relationship between microbial metabolites and allergic diseases and identify functionally important metabolites that affect allergic immune responses.

## Microbial metabolites influence allergic disease development

Allergies, such as hay fever and asthma, are common and can be severe. They often result from the body’s reaction to harmless substances such as pollen or certain foods. This Review explores how gut bacteria and their metabolites influence allergies. Researchers found that people with allergies often have less diverse gut bacteria. This imbalance, known as dysbiosis, can lead to fewer beneficial metabolites such as short-chain fatty acids (SCFAs), which help regulate the immune system. The study reviewed various experiments and clinical data to understand how these microbial metabolites affect allergies. SCFAs, for example, can strengthen the gut lining and reduce inflammation, potentially lowering allergy risks. The research also highlighted the role of other metabolites in managing immune responses. The findings suggest that improving gut health and the immune system through diet,probiotics, and postbiotics might help prevent or treat allergies.

This summary was initially drafted using artificial intelligence, then revised and fact-checked by the author.

## Introduction

Type 1 hypersensitivity-associated allergies occur in response to various environmental and food-derived substances. While these responses are important for host defense by promoting elimination of parasites and immunogenic substances from the body, they have harmful effects on the body, causing rapid life-threatening anaphylaxis, breathing problems, bowel hypersensitivity or chronic inflammatory responses in tissues. In terms of common allergies, allergic rhinitis (AR) is caused by airborne environmental allergens such as pollens, house dust mites (HDM), animal dander, mold spores, cockroach debris and occupational materials^1^. Atopic dermatitis (AD) or eczema is commonly caused by skin exposure to HDM, animal dander from cats and dogs, and pollen^2^. Allergic asthma is caused by inhaled antigens such as pollen, dust mites, pet dander and cockroach proteins^3^. Food allergies (FAs) are frequently caused by exposure to dietary antigens in foods such as peanut proteins (Ara h 1 and Ara h 2), milk proteins (caseins and whey), egg proteins (ovalbumin and ovomucoid) and proteins from shellfish (tropomyosin)^4^.

Excessive exposure to allergens on the epithelial surfaces of the skin, respiratory tract and alimentary tract due to weakened barrier function can promote the development of allergic diseases. Another important factor is weakened or defective immune tolerance to allergens in foods or the environment^5^. One in five children in developed countries experience allergies ranging from mild hay fever and respiratory allergies to more severe anaphylactic allergies^6–9^. Allergic conditions such as rhinitis, asthma, dermatitis and FAs are primarily driven by type 2 cytokines (IL-4, IL-5, IL-9 and IL-13) and allergen-specific IgE^10^. These immune mediators increase the activity of T cells, group 2 innate lymphoid cells (ILC2), mast cells, eosinophils and basophils in affected tissues, leading to the characteristic inflammation and symptoms of these allergies The rise in allergies in developed nations over the past few decades is not entirely clear. However, a mounting body of evidence suggests a role for Westernized lifestyle factors in regulating allergic diseases.

It has been established that diet and gut microbiomes considerably affect the incidence of diverse allergies in animal models and human populations^11–17^. Microbial changes, including reduced diversity and dysbiosis, occur in the gut and affected tissues such as lungs and skin of allergic patients^18–20^. Gut microorganisms process and metabolize dietary materials and host secretions to produce a myriad of metabolites^21^. Major microbial metabolites are made from carbohydrates, proteins and cholesterol metabolites. More specifically, short-chain fatty acids (SCFAs) such as acetate, propionate and butyrate are produced from dietary fibers and resistant starches^22^. Major amino acid metabolites are produced from tryptophan (Trp), arginine (Arg) and tyrosine (Tyr)^23–25^. Primary bile acids (BAs) are made from cholesterol in the liver and are modified (that is, dehydroxylated) by gut microorganisms to become secondary BAs^26^. In addition, there are many other metabolites with positive or negative functions in regulating the host immune system^27,28^.

**Fig. 1:**
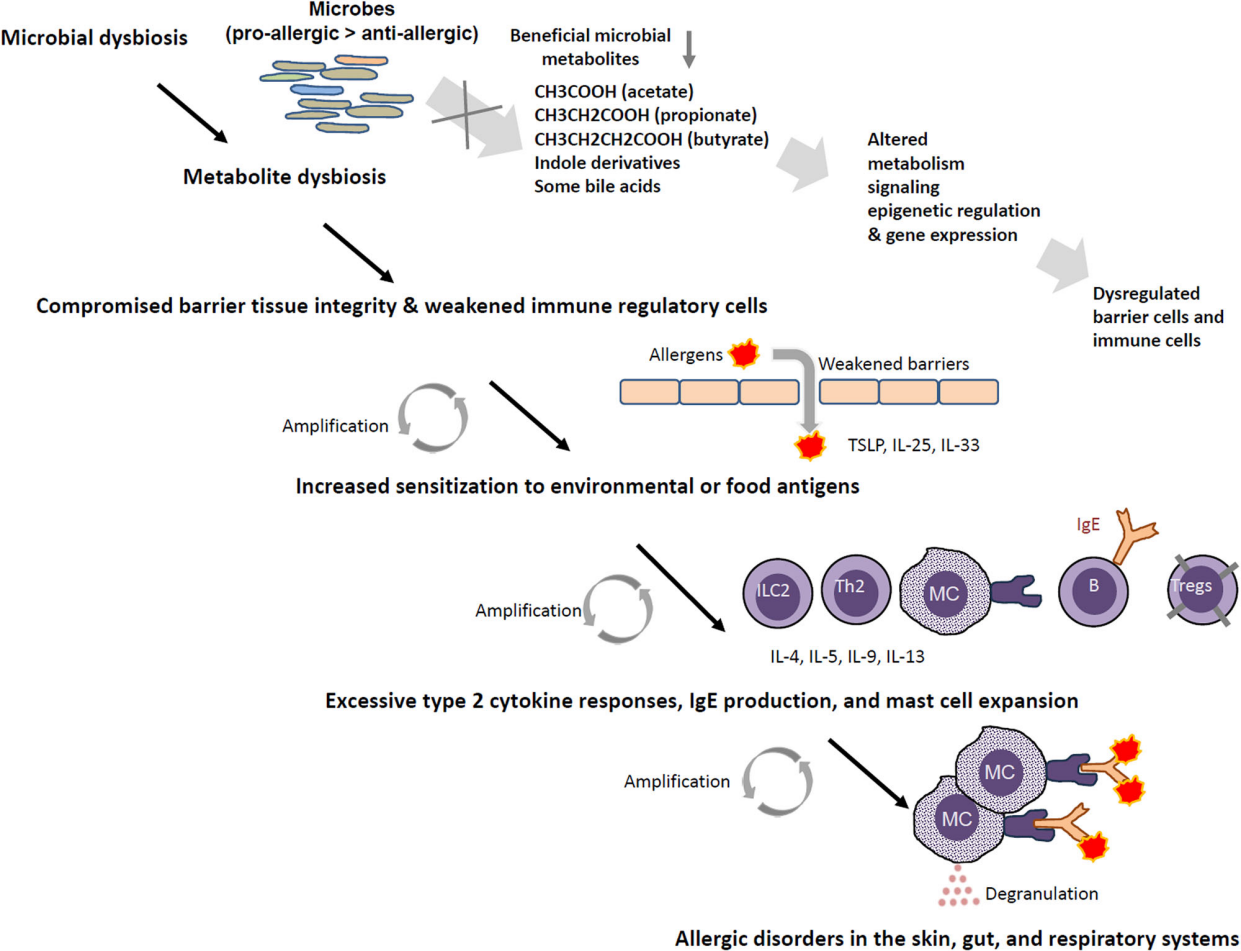
General impact of microbial metabolite dysbiosis on allergic pathogenesis. Microbial dysbiosis is a risk factor, occurring even before the onset of allergy pathogenesis. Microbial dysbiosis accompanies altered production of microbial metabolites, reducing the production of beneficial metabolites such as SCFAs, indole derivatives and BA metabolites, among others. At the same time, the production of harmful microbial metabolites or products from pathogenic microorganisms is increased. These changes weaken the barrier function in the skin, respiratory tract and intestine, increasing environmental or food antigen exposures to the immune system in the tissues. This process is called antigenic sensitization. Due to heightened expression of allergic alarmin cytokines such as TSLP, IL-25 and IL-33, the recruitment and tissue population of immune cells such as ILC2, eosinophils, basophils and mast cells are increased over time. DCs differentiated under these conditions would migrate to secondary lymphoid tissues, where they promote the differentiation of Th2, Tfh and Tc2 cells, which collectively induce allergen-specific IgE production by B cells and drive allergic tissue inflammation. Allergen-specific IgE molecules bind to the high-affinity receptor FcεRI on mast cells for their sensitization and ultimate activation by allergens and other inflammatory signals in tissues. Following repeated exposure to allergens, these processes are eventually amplified to pathogenic levels and cause chronic allergic diseases.

Microbial metabolites affect barrier tissue cells and immune cells via specific receptors^21^. Microbial metabolites also serve as nutrients for tissue cells and immune cells, increasing their cellular energy levels, affecting cellular state, differentiation and functions^29^. These metabolites can also regulate important enzymes such as histone deacetylases (HDACs)^30^. These effects can profoundly affect cellular activities and gene expressions to control barrier cells and immune cells involved in allergic immune responses (Fig. 1). The purpose of this review is to assess and summarize the current research development on the roles of gut microbial metabolites in regulating allergies in experimental and clinical settings.

## Microbial dysbiosis in allergies

**Fig. 2:**
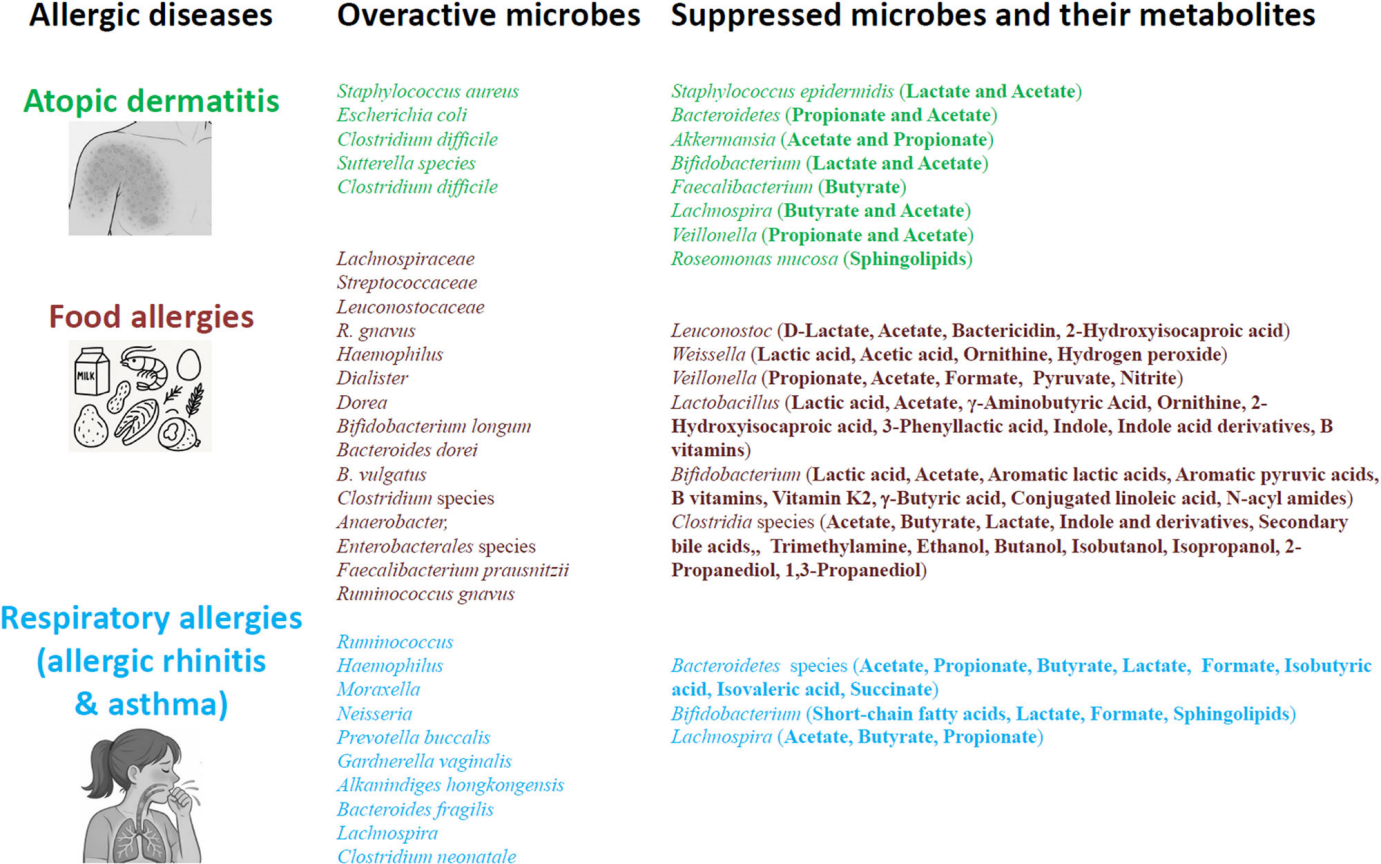
Microbial and metabolite dysbiosis in allergies. In major allergic conditions, the microbiomes in the intestine and affected tissues such as the skin and respiratory system are altered, increasing certain pathogenic strains while decreasing beneficial metabolite-producing microorganisms, leading to microbial metabolite dysbiosis. This can amplify pathological immune responses, leading to persistent allergies. Listed above are the metabolites produced by microbes frequently altered in allergies^182,183–191,192–201,202–207^. While simplified here, the microbes and their metabolites may have complex roles, both exacerbating and ameliorating allergic pathogenesis depending on tissues and diseases.

The full colonization of the human intestine in early life is a gradual process that continues over the first few years after birth^31^. Colonization of the lungs starts to occur at the same time within the second week of life and is mostly completed by the first few years of life^32^. The microbiome is required to support barrier tissue immunity and influences the fitness of the immune system^33^. The gut microbiome is diverse with 500–1,000 species, the size and composition of which can change depending on host conditions and diets^34^. Healthy individuals without allergic diseases or symptoms tend to have a more diverse microbiome in the gut, and this is associated with better working immune systems with high levels of immune tolerance^35,36^. Meanwhile, allergic individuals often exhibit reduced microbial diversity, especially in early life. In microbial dysbiosis, decreased levels of beneficial bacteria and/or increased levels of harmful bacteria occur (Fig. 2). Allergy-associated dysbiosis is thought to be caused, in part, by genetic variations, inflammatory responses in affected tissues, unhealthy lifestyles including poor diet choice, or changes in diet due to allergy symptoms. More specifically, aversion behaviors, occurring after allergic episodes, can contribute to changes in the gut microbiome by altering diet choice and lifestyle.

Microbial colonization of the skin occurs immediately at birth, but the cutaneous microbial community increases its diversity with age over the first year of life^37–39^. The early diversification or maturation of the microbial community is important for lowering the AD incidence in children in later years^40,41^. Thus, delayed maturation or dysbiosis of microbiome can occur in apparently normal babies without any diseases. However, these babies are more likely to develop AD later in life. Microorganisms on the skin and in the gut affect AD pathogenesis. *Staphylococcus aureus* is often the dominant microbial species associated with AD. *S. aureus* overgrowth can disrupt lipid metabolism and compromise the skin barrier. By contrast, colonization of *Staphylococcus epidermidis* and *Roseomonas mucosa* is beneficial in that they inhibit S*. aureus* colonization and affect immune responses to prevent AD development. Also increased in AD are *Escherichia coli*, *Clostridium difficile* and *Sutterella* species. Overgrowth of *C. difficile* and *E. coli* in infants with AD has been linked to decreased beneficial bacteria, abnormal gut barrier function and a loss of immune tolerance^41^. Bacteroidetes, *Akkermansia*, *Bifidobacterium* and *Faecalibacterium* are often found in lower proportions in patients with AD. In general, AD is associated with reduced levels of butyrate-producing bacteria^42^.

FAs manifest in various symptoms in respiratory, digestive and skin tissue systems. Microbial changes also occur in FAs^43,44^. For example, increased Streptococcaceae, Lachnospiraceae and Leuconostocaceae have been observed in children with egg allergies. By contrast, increased levels of Clostridia and Firmicutes are linked to the resolution of milk allergy^45^. However, a caveat is that some Clostridia species, such as *Ruminococcus gnavus*, are positively associated with FA^46–49^. Increased levels of *Dorea*, *Dialister*, *Haemophilus* and *Clostridium* species were found in individuals with FA who were sensitive to milk, egg, peanut, soy and wheat, while *Leuconostoc*, *Veillonella* and *Weissella* were underrepresented in infants who developed FAs later^50,51^. Decreased numbers of Bacteroidetes species were observed in food-sensitized children^52^. Young infants with lower levels of *Lactobacillus* and *Bifidobacterium* species were more likely to develop FAs to egg white, cow’s milk and airborne allergens^53,54^. Decreased abundance of certain bacteria, such as *Bifidobacterium longum*, *Bacteroides dorei* and *Bacteroides vulgatus*, has been observed in allergic patients. By contrast, an increase in *S. aureus* has been linked to FAs to eggs and milk^55^. Increased levels of *Clostridium* and *Anaerobacter* species have been observed in infants with IgE-mediated FA^56^. FA has also been associated with increased abundance of Enterobacterales species^57^. *Faecalibacterium prausnitzii* and *R. gnavus* were increased in patients with FAs as well as respiratory allergies^46^. Despite the association of lower fecal diversity in infancy and allergy incidence, higher microbial diversity is paradoxically associated with increased risk of developing allergic diseases in adults^58^. Therefore, diversity by itself is not a strong indicator of allergic diseases later in life. A fecal microbiome analysis in healthy and food-allergic twins revealed increased abundance of *Phascolarctobacterium faecium* and *Ruminococcus bromii* in healthy versus FA twins^59^. Importantly, *Phascolarctobacterium faecium* produces SCFAs, and *Ruminococcus bromii* can process resistant starch^60,61^.

In the lungs, it is estimated that healthy individuals have 10^3^–10^5^ bacteria per gram of the tissue, largely composed of Proteobacteria, Firmicutes, and Bacteroidetes species. Early disruptions in the intestinal microbiome can potentially increase the risk of developing AR later in life^62^. Microbial dysbiosis in the gut may exacerbate nasal inflammation and rhinitis symptoms. It has been reported that *Ruminococcus* species were enriched in a mouse model of AR compared with control animals. It appears that the microbial changes associated with asthma are highly diverse depending on cohort^63^. At this point, the impact of many of these microbial groups or species on respiratory allergies remains largely speculative.

Asthma subtypes include neutrophilic, eosinophilic, mixed granulocytic and paucigranulocytic asthma, depending on histological features. It is also classified as allergic, non-allergic, occupational, seasonal and exercise-induced depending on triggers and disease modes. Eosinophilic and neutrophilic asthma are relatively strongly linked to changes in the airway microbiome^64,65^. In patients with asthma, there is a shift in the lung microbiota toward increased diversity, while the gut microbiota shows decreased diversity^66,67^. For example, the Proteobacteria group was increased in abundance in the respiratory system of asthma patients. More specifically, *Haemophilus*, *Moraxella* and *Neisseria* species have been associated with more frequent viral infections and severe asthma symptoms^68^. *Prevotella buccalis*, *Alkanindiges hongkongensis* and *Gardnerella vaginalis* were overrepresented in the nasal microbiota of individuals with asthma compared to controls. Colonization with *Bacteroides fragilis* strains has been proposed as a potential predictor of possible asthma development^11,69^. The bacterial genera *Veillonella*, *Faecalibacterium*, *Lachnospira* and *Rothia* were underrepresented in fecal samples of 3-month-old children with asthma^70^. Early colonization with these microorganisms was associated with increased blood eosinophil counts and total IgE levels^71^. Reduced intestinal abundance of Bacteroidetes and *Bifidobacterium* was also observed in those with allergic airway diseases^72^. A decreased abundance of *Lachnospira* species and *Clostridium neonatale* in infants’ stool was correlated with asthma development at 4 years of age^73^, whereas *C.* *difficile* colonization was associated with allergy development later in life^74^.

## Major microbial metabolites and their impact on host cells

The normal gut microbiome, composed of approximately a thousand species, can produce a myriad of metabolites when there are enough nutrient materials in the colon. Because each microbial species has a finite number of genes, it can produce only a limited range of metabolites. In the case of dysbiosis, where a small number of microbial species become dominant, this can substantially limit the production of certain metabolites. Moreover, diet and health status are key regulators of microbiomes and help determine the mass and diversity of microbial metabolites. In most allergic conditions, reduced or altered production of SCFAs has been observed^75,76^. Moreover, alterations in the metabolism of Trp and BAs are linked to dysregulated immune responses in allergic individuals^77–79^.

Carbohydrates that reach the colon are degraded and fermented by the microbiome to SCFAs^80,81^. Because starches are mostly digested and absorbed in the upper alimentary tract, it is mainly the hard-to-digest complex carbohydrate polymers, such as dietary fibers and resistant starches, that are available for microorganisms to make SCFAs in the colon. Butyrate, propionate and acetate are the major SCFAs in the mammalian colon. SCFAs regulate cells by several different pathways. The first way to affect host cells is by activating G-protein-coupled receptors (that is, GPR41, GPR109A, GPR43 and Olfr78) on host cells^80,81^. This activation will trigger Gαi/o proteins (GPR41 and GPR109A) and Gαq/11 and Gαi/o proteins (GPR43)^82–84^. Second, SCFAs are used as an energy source after transportation into cells and conversion to acetyl-CoA^29^. SCFAs can also affect metabolic pathways such as glycolysis and oxidative phosphorylation. The third way to regulate host cells is to inhibit type 1 and 2 HDACs for gene expression regulation^85,86^. SCFAs regulate both innate and adaptive immune cells, including macrophages, dendritic cells (DCs), ILCs, B cells and T cells^29,86–89^. More specifically, SCFAs increase IL-10 production to promote immune tolerance and enhance immunity by boosting the numbers of effector T cells, including Th1, Th17 and effector CD8^+^ T cells, in the intestine^80,86^. SCFAs increase antibody production by inhibiting HDACs and metabolically support B cell differentiation to IgA- or IgG-producing cells^29^. SCFAs increase the activity of both ILC2 and ILC3 during active immune responses^88^. It is also reported that SCFAs suppress ILC2 in the lungs during experimental asthma development^90^.

Primary BAs are made in the liver from cholesterol and play important roles in digestion and nutrient absorption, particularly of fats and fat-soluble vitamins^91^. Primary BAs are typically conjugated with amino acids such as glycine or taurine in the liver. They are stored in the gallbladder before secretion into the duodenum. In the colon, they are modified by gut microorganisms to become secondary BAs. The modifications include deconjugation (removing glycine or taurine), dihydroxylation, oxidation and epimerization^92^. Major primary BAs are cholic acid and chenodeoxycholic acid. The most abundant secondary BAs in the intestine are deoxycholic acid (DCA) and lithocholic acid (LCA)^93^. Both primary and secondary BAs are reabsorbed into the ileum and returned to the liver. Farnesoid X receptor (FXR) and the Takeda G-protein-coupled receptor 5 (TGR5) are the major receptors for BAs^91^. FXR is highly expressed by hepatocytes, enterocytes, pancreatic β-cells, kidney cells, adrenal gland cells and immune cells such as macrophages, DCs and natural killer T cells^94^. TGR5 is highly expressed by the gallbladder epithelium, bile duct cholangiocytes, intestinal stem and epithelial cells, Kupffer cells, liver sinusoidal endothelial cells and neuron cells^95–99^. Among immune cells, TGR5 is expressed by monocytes and macrophages. Other major receptors for BAs include pregnane X receptor (PXR) and S1PR2^100,101^. Within the immune system, BAs control gut microorganisms with their antimicrobial activity, support the intestinal barrier function by regulating epithelial cells, and regulate macrophages and DCs for their functions in cytokine production and T cell activation^102,103^. TGR5 activation by DCA and LCA decreases the production of IL-12 and TNFα, and FXR activation by isoallo-LCA promotes T_reg_ cells at the expense of Th1 and Th17 cells^104^.

Amino acids are also degraded and modified by microorganisms. Among them, microbial metabolites from Trp (indole derivatives), Arg (nitric oxide and ornithine), branched-chain amino acids, Phe (for example, phenylacetylglutamine and phenylacetic acid) and Tyr (tyramine, 4-hydroxyphenylpyruvate and *p*-cresol) have been known to have important immune regulatory functions^21^. Indole metabolites regulate cells via the aryl hydrocarbon receptor (AhR)^105^. AhR activation by indole derivatives can affect Th1 versus Th2 immune responses, potentially suppressing Th2 polarization in allergic immune responses^106^. Ornithine is a precursor of polyamines, which have tissue-repair and anti-inflammatory properties^107^. Phenylacetylglutamine has been shown to activate α2A, α2B and β2-adrenergic receptors^108^. Phenylacetic acid is implicated in promoting AhR signaling^109^. Phenylpropanoic acid activates GPR109B, and cadaverine triggers HRH4^110^.

## Key immunological features that affect allergic immune responses in the skin, respiratory and intestinal tissues

**Fig. 3:**
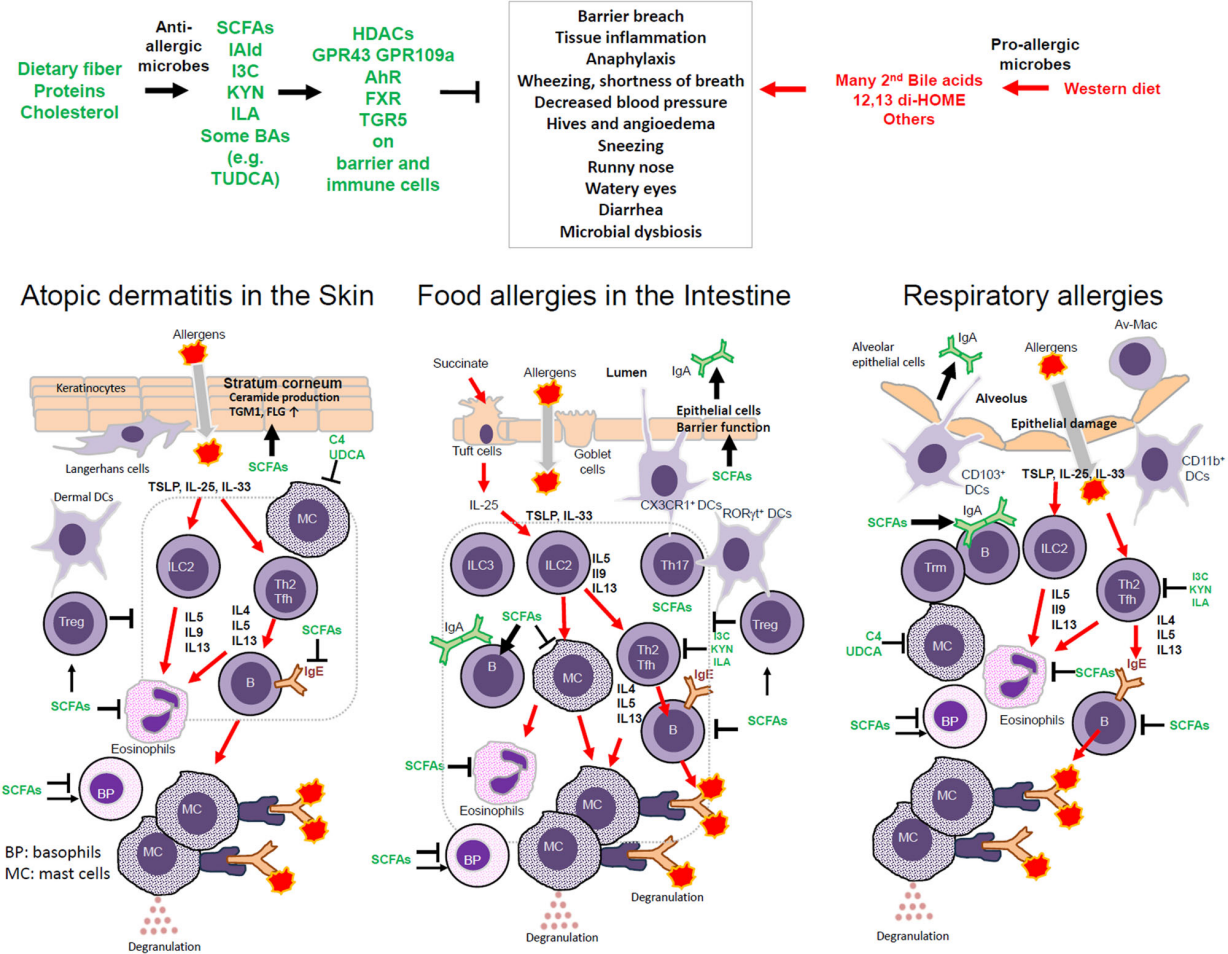
Regulation of immune cells by microbial metabolites in the skin, intestine and lungs. The three barrier tissues or organs have shared yet distinct structures and cell compositions. The skin immune system consists of multiple layers of keratinocytes containing LCs, with underlying layers housing other immune cells such as dermal DCs, ILC2s, T cells, mast cells and others. The intestinal immune system is composed of a single layer of epithelial cells in villi in the small intestine and crypts in the colon at various differentiation stages or fates for distinct functions. Tuft cells that sense succinate from microorganisms and goblet cells that produce mucin and transport protein antigens from the lumen are good examples. The intestine has T cells in the epithelial layer (i.e., intestinal epithelial lymphocytes) and lamina propria. ILC3 plays a critical role in supporting epithelial barrier function and immunity, which are also supported by Th17 cells. Specialized DCs such as CX3CR1^+^ DCs can sense the presence of luminal antigens, and RORγt^+^ DCs (also called Thesis cells) are important for the generation of RORγt^+^ FoxP3^+^ T cells that protect the intestine from developing inflammatory diseases. B cells actively produce IgA, which is secreted to the intestinal lumen to bind commensal bacteria and potential pathogens. Lungs are composed of alveoli that are formed by a single layer of type I AECs (also called pneumocytes) and type 2 AECs that function as stem cells and secrete surfactants. AMs patrol the alveolar space to clean up any particles in the breathed air. ILC2 are the dominant ILCs in the lungs. Several DC types and macrophages monitor antigens in the alveolar and interstitium. T cells, DCs and ILCs are in the interstitium and mucosal-associated lymphoid tissues in the lungs. The presence of T_reg_ cells is important to keep these tissues from developing excessive inflammatory responses. Upon barrier breach due to genetic, physical and chemical reasons, inflammatory cytokines such as TSLP, IL-25 and IL-33 are produced to initiate pro-allergic Th2 type inflammatory responses characterized by IL-4, IL-5, IL-9 and IL-13. Due to compromised barrier functions, allergens are introduced and captured by antigen-presenting cells such as DCs, which migrate to secondary lymphoid tissues to activate and differentiate T cells. Because of the cytokine milieu rich in type 2 cytokines, allergen-specific Th2 cells are generated in increased numbers. Th2 cells further increase the production of IL-4, IL-5, IL-9 and IL-13, which skew B cell differentiation into IgE producers. The cytokines increase the numbers of mast cells, basophils and eosinophils, and allergen-specific IgE sensitizes them by binding to the FcεRI complex on mast cells. Whenever there are allergen challenges through the weakened barriers, these allergic granulocytes undergo activation to degranulate or secrete a battery of allergy mediators such as histamine, neutral proteases (tryptases and chymases), proteoglycans (heparin), cytokines (TNFα, IL-4, IL-6, IL-13 and TSLP) and lipid mediators (prostaglandins and leukotrienes). Moreover, mast cells are activated by various inflammatory stimuli, such as changes in pressure, temperature, complements, IgG, pathogen-associated molecule patterns, neuropeptides, cytokines (SCF, IL-33, IL-25 and TSLP) and extracellular ATP in tissues. Microbial metabolites have several different regulatory effects on allergic immune responses. First, the barrier cells are strengthened for their functions by microbial metabolites such as SCFAs. Second, T and B cells are regulated by microbial metabolites such as SCFAs and indole metabolites to produce protective T_reg_ cells and IgA, but not Th2 and IgE. Moreover, the allergy-associated granulocytes, including mast cells, are regulated by the microbial metabolites especially by SCFAs and TUDCA/UDCA. Other BAs have generally stimulating effects on immune cells.

The skin, lungs and intestines possess unique immune systems tailored to their specific needs and microbial exposures. These organs have unique combinations of tissue and immune cells and develop general as well as tissue-specific immune responses, which may lead to pathological allergic conditions (Fig. 3).

The skin is a bona fide barrier tissue with keratinocytes in the epidermis, sweat glands and hair follicles. The epidermis acts as the first line of defense in the skin, producing antimicrobial peptides and communicating with other immune cells through cytokines and chemokines^111^. It is also activated by pathogenic microorganisms to initiate allergic immune responses. For example, *S. aureus* induces IL-33 production in keratinocytes^112^. Langerhans cells (LCs), a specialized type of dendritic cell (DC), are present in the epidermis. LCs are regulated by host and microbial metabolites. The indole derivative, indole-3-aldehyde (IAId), activates AhR and suppresses the function of LCs, leading to IL-10 production and decreased CD4^+^ T cell proliferation^113^. In addition, there are conventional DCs in the dermis layer (dDCs). These dDCs capture and present antigens to T lymphocytes after migration to lymphoid tissues. The skin is a nonlymphoid tissue and therefore harbors only memory, effector or tissue-resident, but not naive, types of T cells. In particular, a significant number of memory T cells, including CD8^+^ tissue resident memory T cells (called T_RM_ cells) reside in the epidermis, whereas many CD4^+^ T cells are found in the dermis^114^. These cells provide rapid and localized immunity upon reinfection. dDCs also play a role in antigen presentation and support T cell responses^115^. The majority of dDCs are IRF4^+^ DC2, which can readily induce Th2 cells, a property shared with LCs. In addition, basophils, ILC2, mast cells and eosinophils, which are found in the dermis of the skin, can also promote Th2 cells in the skin^116^.

The lungs also have unique cell types such as alveolar macrophages (AMs) and airway epithelial cells (AECs), which form a complex tissue network embedded with innate immune cells^117^. The mucociliary system traps and clears pathogens in the upper respiratory tract. AECs and AMs secrete antimicrobial peptides, cytokines and chemokines, initiating a rapid, nonspecific response to pathogens. These cells express Toll-like receptors, which recognize microbial components and trigger immune cell activation and cytokine production. The lungs have many ILCs in the adventitial cuffs around airways and vessels in the alveolar capillary beds, among which ILC2 is the dominant cell type^118–121^. The lungs also have T lymphocytes, IgA-producing B lymphocytes and specialized DCs^122^. T_RM_ cells in the lungs can mount rapid responses to previously encountered pathogens^123^. The role of IgA in neutralizing pathogens in the airways is important in the respiratory tract^124^. The propensity to develop Th2-type allergic immune responses in the lungs is probably largely due to the nature of the antigens (for example, pollen, dust mites, animal dander and fungal spores), Th2-biased epithelial alarmin responses (TSLP, IL-25 and IL-33) and the activity of ILC2s. It has been shown that SCFAs have positive effects on fighting respiratory infections^125^.

Unlike the skin, the intestine has a single layer of epithelial cells that serves as a barrier and is responsible for processing food into absorbable nutrients. Therefore, there is a need to develop stronger immune tolerance to food antigens. Another factor is the high load of gut microorganisms in the colon as well as the ileum. The microorganisms are part of the digestion system to process hard-to-digest materials to extract additional nutrients and synthesize certain metabolites important for the host. The intestinal microbiome is significantly greater than those on the lungs and skin in metabolite production, leading to higher and more diverse production of metabolites. These metabolites are transported via blood vessels to most parts of the body, including the skin and lungs. An important task of intestines is to maintain a balance between defending against pathogens and tolerating food antigens and commensal microbiota. Intestinal epithelial cells produce enzymes, defensins and mucins^126^. In particular, antimicrobial peptides are produced by Paneth cells, which are specialized epithelial cells in the crypts. The intestine has gut-associated secondary lymphoid tissue such as Peyer’s patches and colonic patches for activation of T and B cells. As in the lungs, IgA is an important immunological factor for the intestine, binding and keeping pathogens and the gut microbiome from invading^127^. ILCs play important roles in directing intestinal epithelial cells to form a strong physical and chemical barrier by inducing the expression of epithelial tight junction proteins and antimicrobial peptides^128^. The intestine is rich in production of IL-33, IL-25, TSLP, IL-4, IL-9, IL-5 and IL-13 by epithelial cells, T cells, basophils, ILC2 and/or Tuft cells^129^. Activation of this pathway is key in allergic immune responses in the intestine. The intestinal epithelial cells require microbial metabolites for the maintenance of intestinal barrier and immunological functions^29,86,87,130^. Intestinal Tuft cells are activated by succinate, produced by parasites, such as *Polymorphus minutus* and *Trypanosoma brucei*. This is mediated by the succinate receptor 1 (SUCNR1) on Tuft cells to produce IL-25, which promotes ILC2 expansion^131^. Butyrate inhibits the activity of HDAC3, a HDAC enzyme that is crucial for stem cell differentiation into Tuft cells^132^. Thus, succinate and butyrate can differentially regulate type 2 immunity to combat parasites, which could be dysregulated to promote allergic immune responses.

## Regulation of AD by microbial metabolites

Microbial metabolites, produced by bacteria in the skin and gut, play a significant role in regulating AD pathogenesis. In particular, SCFAs and indole derivatives can modulate immune responses, lipid metabolism and skin barrier function, impacting AD development and severity^133^.

SCFAs are believed to reach the skin through the bloodstream or to be produced locally within the skin. Propionate is a sebum-derived metabolite produced in the skin^134^. Butyrate can be metabolized by keratinocytes to produce acetyl-CoA. SCFAs promote the production of ceramides, which are utilized to strengthen the skin barrier^135^. Moreover, SCFAs promote keratinocyte metabolism and differentiation and help form the stratum corneum (that is, the outside layer of the skin)^135^. SCFAs can support the immune cells in the skin, such as macrophages, ILCs, T cells and B cells^29,86,88,136^. SCFAs can inhibit the inflammatory response triggered by inflammatory skin bacteria such as *Propionibacterium acnes*^137^. HDAC inhibition by butyrate increases the expression of transglutaminase-1 (TGM1) and filaggrin (FLG)^138^. SCFAs can increase cellular metabolism including mitochondrial tricarboxylic acid cycle for energy production in skin cells^29,135^. Thus, SCFAs, whether produced in the skin or the intestine, can strengthen the skin barrier and help maintain skin immune homeostasis. This can decrease the sensitization of the skin immune system by various environmental antigens. SCFAs have anti-inflammatory effects, and SCFA production is associated with a lower risk of AD^139^. In contrast to the largely protective roles of SCFAs, the level of six-carbon SCFA caproic acid was increased in patients with AD^139^. Thus, caproic acid may have a proinflammatory effect on the skin.

The level of IAId, a tryptophan metabolite that triggers AhR, was found to be significantly decreased on both lesional and nonlesional skin of patients with AD^140^. IAId is produced by skin commensal bacteria, and it has been shown to negatively regulate skin inflammation in patients with AD. A related AhR ligand, indole-3-carbinol (I3C), has been shown to reduce skin inflammation and the levels of allergen-specific IgE and TSLP^141^.

Dermatitis development is associated with elevated levels of BAs in the skin, which can further contribute to inflammation and pruritus (that is, itch)^142^. BA secretion is increased in people consuming a Western style diet with high levels of fat and meat and low levels of dietary fibers, and this is associated with an increased incidence of dermatitis with features of Th2 and Th17 inflammation, both of which are involved in AD pathogenesis^142^. BA sequestrants, such as cholestyramine, when used topically, have been shown to reduce epidermal thickening and levels of cutaneous inflammatory cytokines in a mouse model of dermatitis^142^. While BAs are associated with increased AD symptoms, the secondary BA ursodeoxycholic acid (UDCA) appears to alleviate AD-associated inflammation^143^. More specifically, UDCA decreases the activities of MAPK and NF-kB and the production of MCP-1, MDC, TARC and IL-6 by keratinocytes and mast cells. In addition, a microbial metabolite associated with atopy is 12,13-diHOME (12,13-dihydroxy-9Z-octadecenoic acid), a fatty acid derivative produced by microorganisms that can promote macrophage expression of inflammatory cytokines (IL-1β, TNFα and IL-6), B cell proliferation and IgE production^144^.

## Regulation of FA by microbial metabolites

FA incidence has risen to high levels in the past several decades in developed countries, with 5–8% of the population affected depending on the country and age group^145^. Children have a higher incidence of FA than adults. The high prevalence of this disorder in developed countries and in younger populations implies the potential importance of diet, the microbiome and immunological maturation. Patients with FA react to certain foods (including the nine most common allergens: peanuts, nuts, milk, eggs, wheat, soy, sesame, crab and shrimp) that are introduced to multiple organs such as the gastrointestinal tract, respiratory system and skin^146^. The etiology of FA remains unknown, but it is caused by overzealous or excessive immune responses to food-derived protein antigens, which are primarily mediated by type 2 immune responses involving Th2 cells, IgE production and mast cell activation. Defective barrier functions in the skin and the intestinal tract are also suspected to increase lymphocyte activation in response to food antigens^147,148^.

Studies have linked low SCFA levels in early life to an increased risk of FA. In particular, butyrate has shown promise in protecting against FA. Butyrate strengthens the intestinal barrier in part by suppressing stress-mediated Notch signaling^149^. The levels of major SCFAs in feces were significantly reduced in allergic individuals^43^. *P. copri*, a producer of acetate and propionate, was highly increased in the healthy group compared with the FA group. *P. copri* was generally increased in the gut when a diet rich in fibers was consumed^150^ or in Th1-associated diseases such as colitis and rheumatoid arthritis^151,152^. Another group reported decreased butyrate in allergic patients, and oral administration of butyrate ameliorated some of the FA-associated immune responses^149^. Butyrate decreased ovalbumin-induced experimental anaphylaxis in mice. Another important function of butyrate is to suppress IgE-mediated mast cell activation^153^. HDAC inhibition is a mechanism behind the suppression of mast cells by butyrate. The signaling molecules upon triggering of the IgE receptor FcεR1, such as Bruton tyrosine kinase (BTK), spleen tyrosine kinase (SYK) and linker for activation of T cells (LAT), were found to be downregulated by butyrate in their expression in mast cells. HDAC inhibition can increase histone acetylation and boost the expression of many genes. Another study found that SCFAs activated GPR109A (a receptor for butyrate and niacin) on mast cells and increased the expression of prostaglandin E2 (PGE2), leading to inhibition of mast cells^154^. Moreover, SCFAs can contribute to the development and function of regulatory T cells to support oral tolerance to food antigens^155^.

Indole metabolites are also linked to suppressed FAs. *Bifidobacterium* *breve* M-16V promotes the production of indole derivatives such as indole-3-propionic acid (IPA) by other microorganisms, and its supplementation ameliorated cow’s milk allergy^156^. In this regard, IPA supplementation decreased allergy symptoms, which was mediated by AhR activation by IPA. Another ligand of AhR, I3C, which is abundant in cruciferous plants, decreased ovalbumin-specific IgG1 and peanut allergy symptoms^157^.

Primary BAs, including chenodeoxycholic acid, can increase immunological sensitization to food antigens. This appears to be mediated by increasing the expression of retinoic acid-regulated genes and the production of food allergen-specific antibodies (IgE and IgG1)^79^. More studies are required to better understand the interaction and functions of BA and retinoic acid pathways. Peanut oral immunotherapies have been developed to treat patients with peanut allergy. Mixtures of primary and secondary BAs (that is, cholic acid, chenodeoxycholic acid and UDCAs) support colonic FOXP3^+^ T_reg_ cells expressing the transcription factor RORγ^158^. These T_reg_ cells have the potential to suppress inflammatory immune responses^159^. A specific profile of fecal BAs before tolerance-inducing treatment predicted the efficacy of the oral peanut immunotherapy^160^. Thus, BAs have complex functions in regulating intestinal immune responses including those for FAs. An interesting feature of the microbiome or microbial metabolites associated with oral immunotherapy failure is the increased amino acid utilization, which could degrade and therefore decrease amino acid-conjugated secondary BAs. Certain BA-producing microbial species such as *R. gnavus* were elevated in food-allergic patients who were responding to oral immunotherapy^46^. Despite some exceptions, many BAs appear to exert pro-allergic effects on FA pathogenesis.

## Impact of microbial metabolites on respiratory allergies

It has been reported that SCFAs, particularly butyrate and propionate, can protect people from developing asthma. Butyrate downregulated GATA3 expression and suppressed ILC2-driven airway hyperresponsiveness^161^. Studies have linked decreased levels of fecal SCFAs in infants to increased risks of developing asthma^161–164^. Increased levels of butyrate and propionate in baby stools have been associated with decreased asthma development^76^. SCFAs regulate immune responses by activating GPCRs and inhibiting HDACs^86,87^, which impact inflammation, remodeling and hyperresponsiveness in airways. SCFAs regulate the proliferation and function of ILC2, reducing the production of asthma-related cytokines such as IL-5 and IL-13^161^. SCFAs induce the differentiation of T_reg_ cells and suppressive myeloid cells for suppression of allergic airway diseases^86,165^. In addition, SCFAs suppress Th2 cell activity by altering DC function and migration, making them less inflammatory and more tolerogenic^166^. It has been reported that SCFAs can reduce the survival, adhesion and migration of eosinophils, further contributing to the attenuation of airway inflammation^167^. Intestinal helminth infection unexpectedly reduced the severity of allergic airway inflammation in part by increasing SCFA production^168^. Helminth infection altered the intestinal microbiome to increase SCFA production, and SCFAs increased T_reg_ activity in GPR41-dependent manner. Another function of SCFAs is to suppress asthma, in part, by decreasing IL-13-producing Tfh cells that skew B cell production of IgE^169^. Yet, another function of butyrate is to suppress mast cell activation^153^. Moreover, *F. prausnitzii* administration increased *Lachnoclostridium* abundance and SCFA production, alleviating HDM-induced allergic asthma^170^.

Reduced levels of SCFAs were found in patients with AR^75^. Antibiotics, such as vancomycin, can decrease SCFA-producing bacteria and fecal SCFA levels in mice, exacerbating AR severity^171^. Administration of butyrate in a conjugated form to starch increased colonic butyrate concentration, decreased serum allergen-specific-IgE, IL-4 and IL-5, and improved clinical activity in a mouse model of AR^172^. These results indicate that microbial SCFAs are effective suppressors of AR.

Reduced levels of Trp have been associated with increased expression of TSLP and subsequent asthma inflammation^173^. In a mouse model of ovalbumin-induced asthma, the disease severity was alleviated by administration of Trp metabolites, such as kynurenine (KYN), indole-3-lactic acid (ILA), indole acetic acid (IAA) and I3C^174^. Another indole derivative, IPA, reduces mitochondrial respiration and superoxide production in lung tissues, which decreases allergic airway inflammation^175^. Trp metabolites, such as I3C, KYN and ILA, also reduced allergic asthma in a mouse model^174^. These metabolites activate AhR, which can influence the Th1/Th2 balance, favoring Th1-dominant responses and potentially suppressing Th2 responses^106^. The aminos acid Tyr has also been linked to the regulation of lung inflammation in allergic asthma. Increased *p*-cresol sulfate (a Tyr metabolite), generally considered a toxic metabolite, is unexpectedly associated with low asthma risks in children^176^.

BAs are also implicated in asthma development. Specific BAs, such as glycocholic acid (a glycine conjugate of cholic acid) and glycoursodeoxycholic acid (a glycine conjugate of UDCA), were increased in individuals with asthma^177^. Tauroursodeoxycholic acid (TUDCA) can act on airway cells and attenuate allergen-induced airway inflammation (that is, IgE production, mucus secretion, IL-4, IL-5 and IL-13) and lung hyperresponsiveness^178^. This was studied using an HDM-induced airway inflammation model. The mechanism appears to be mediated by inhibiting the unfolded protein response, a cellular process activated under stress conditions such as inflammation. Thus, BAs are ambivalent in regulating asthma responses, either promoting or suppressing asthmatic activity depending on the type of BAs.

## Concluding remarks

The information discussed in this review highlights the strong association between allergic diseases and microbial dysbiosis. Microorganisms, such as *S. aureus* in the skin, Proteobacteria in the lungs and Bacteroidales in the intestine, which are overrepresented in allergic diseases are generally pathogenic. Typically, Clostridiales, *Bifidobacterium* and *Lactobacillum* are decreased in most major allergy diseases, and these microorganisms are thought to produce beneficial SCFAs, BAs and/or indole metabolites. The first few years of life after birth seem to be critical in the maturation of the immune system and prevention of microbial dysbiosis and allergic diseases later in life. The information discussed in this review raises the question of whether various allergic disorders are treatable with intervention strategies using dietary modifications, prebiotics, probiotics and microbial metabolites.

Although many microorganisms have been identified as altered in adults or infants at increased risk of developing allergies, it remains necessary to pinpoint the functionally important species that, if administered, could reduce allergy pathogenesis. While certain probiotic strains have been shown to ameliorate experimental allergies, it is important to identify natural microorganisms that can suppress allergy symptoms in a sustained manner. Identifying specific microbial species and their metabolites that most effectively promote immune tolerance is also crucial for developing targeted therapeutic strategies.

It is exciting to witness the development of new allergy therapies, including antigen-based desensitization immunotherapies that induce antigen-specific T_reg_ cells, monoclonal antibodies targeting IL-4, IL-5 or IL-13, and BTK inhibitors that act on B cells and mast cells^179^. However, these approaches are limited in efficacy or require ongoing use to be effective. More definitive or permanent approaches to stop allergic diseases will require the immune system to gain stable tolerance against allergens. Microbial therapies, including microbial metabolite approaches, hold promise in making these approaches effective. Perhaps certain microorganisms and metabolites may be used as an adjunct strategy to increase the efficacy and duration of therapeutically induced immune tolerance. Microbial metabolites are potentially good approaches to decrease allergic immune responses or train immune cells not to become allergic. In this regard, concentrations and combinations of metabolites that are effective in treating or preventing allergic patients need to be experimentally determined in human participants. There are expected side effects of utilizing microorganism- or metabolite-based therapies. Major examples are potential infections and inflammatory responses, particularly in the urogenital system^180,181^. These issues should be carefully considered when devising therapeutic strategies with microbial or microbial metabolite-based therapies.
